# Presepsin as a diagnostic marker of sepsis in children and adolescents: a systemic review and meta-analysis

**DOI:** 10.1186/s12879-019-4397-1

**Published:** 2019-08-30

**Authors:** Seo Hee Yoon, Eun Hwa Kim, Ha Yan Kim, Jong Gyun Ahn

**Affiliations:** 1 Department of Pediatrics, Severance Children’s Hospital, Yonsei University College of Medicine, 50-1 Yonsei-ro, Seodaemun-gu, Seoul, 03722 Korea; 2 Biostatistics Collaboration Unit, Department of Biomedical Systems Informatics, Yonsei University College of Medicine, Seoul, Korea

**Keywords:** Sepsis, Presepsin, Diagnosis, Meta-analysis, Child, Sensitivity, Specificit

## Abstract

**Background:**

Early diagnosis of sepsis in pediatric patients is vital but remains a major challenge. Previous studies showed that presepsin is potentially a reliable diagnostic biomarker for sepsis in adult and neonates. However, there is no pooled analysis of its efficacy as a diagnostic biomarker for sepsis in children. The aims of the present meta-analysis were to assess the overall diagnostic accuracy of presepsin in pediatric sepsis and compare it to those for C-reactive protein (CRP) and procalcitonin (PCT).

**Methods:**

A systematic literature search was performed in Medline/Pubmed, Embase, the Cochrane Library, and ISI Web of Science to identify relevant studies reporting the diagnostic accuracy of presepsin in patients with pediatric sepsis. Sensitivities and specificities were pooled by bivariate meta-analysis. Heterogeneity was evaluated by *χ*^2^ test.

**Results:**

We identified 129 studies in total. Most were disqualified on the basis of their titles/abstracts and duplication. Four studies were included in the final analysis. They comprised 308 patients aged between 1 mo and 18 y. The pooled diagnostic sensitivity and specificity of presepsin were 0.94 (95% confidence interval [CI]: 0.74–0.99) and 0.71 (95% CI: 0.35–0.92), respectively. The pooled diagnostic odds ratio, positive likelihood ratio (LR), and negative LR of presepsin were 32.87 (95% CI: 2.12–510.09), 3.24 (95% CI, 1.14–12.38), and 0.08 (95% CI, 0.01–0.74), respectively. Heterogeneity was found in both sensitivity (χ^2^ = 11.17; *P* = 0.011) and specificity (χ^2^ = 65.78; *P* < 0.001). No threshold effect was identified among the studies (r = − 0.938). The pooled sensitivity of presepsin (0.94) was higher than that of CRP (0.51) and PCT (0.76), whereas the overall specificity of presepsin (0.71) was lower than that of CRP (0.81) and PCT (0.76). The AUC of presepsin (0.925) was higher than that of CRP (0.715) and PCT (0.820).

**Conclusion:**

Currently available evidence indicates that presepsin has higher sensitivity and diagnostic accuracy, but lower specificity, than PCT or CRP in detecting sepsis in children. However, these results must be carefully interpreted as the number of studies included was small and the studies were statistically heterogeneous.

**Electronic supplementary material:**

The online version of this article (10.1186/s12879-019-4397-1) contains supplementary material, which is available to authorized users.

## Background

Sepsis is a life-threatening condition. It is the leading cause of death or morbidity in the pediatric population [1, 2]. The epidemiology of pediatric sepsis varies [3], but an estimated 1.2 million children worldwide are stricken with it each year [4]. Recent U.S. studies indicated that > 70,000 children were hospitalized for sepsis at a cost of ~$5.0 billion and a mortality rate of ≤20% [1, 5].

However, there has been limited research on pediatric sepsis. Management of this condition has often been adapted from guidelines for adult sepsis [6]. It is difficult to define sepsis in the pediatric patient because of age-dependent vital signs. Moreover, the seriousness of their condition is often unclear [7]. Sepsis in children is usually diagnosed on the basis of systemic inflammatory response syndrome (SIRS) and a suspected or proven infection [7]. Infection is confirmed when the blood culture/stain/PCR results are positive for a specific pathogen. Sepsis is suspected on the basis of clinical, radiological, or laboratory findings [7–9]. More recently, sepsis was defined as a life-threatening organ dysfunction resulting from the deregulation of the host response to infection [10]. Nevertheless, this criterion was formulated for adult sepsis patients. Thus, additional guidelines that reflect age-specific sepsis and stratify its risks for children are necessary [10]. Blood culture remains the gold standard for the confirmation of sepsis [11, 12].

On the other hand, blood culture has considerable limitations such as prolonged time-to-result and false negativity [13]. Delays in administration of the appropriate antibiotics are associated with significant increases in mortality and morbidity [14]. Therefore, biomarkers may play a vital role in the timely diagnosis and management of sepsis [15]. The most widely investigated diagnostic biomarkers for pediatric sepsis are C-reactive protein (CRP) and procalcitonin (PCT) [16]. However, CRP alone lacks the specificity to discriminate bacterial, viral and noninfectious inflammatory conditions [17]. The prediction of sepsis is also inaccurate as its sensitivity is low [17, 18].

PCT is a promising diagnostic marker for sepsis [11, 19–21] but is inadequate for pediatric sepsis prediction because its sensitivity and specificity are variable [11, 22]. The large discrepancies between studies in terms of their reported cutoff values limit the utility of these biomarkers in clinical practice [20]. After the onset of infection or inflammation, CRP increases within 4–6 h and peaks at 36–72 h [17, 23], while PCT rises within 2–4 h and reaches its maximum at 24–36 h [24–27]. Thus, extra care is necessary when using CRP and PCT as very early biomarkers for sepsis.

CD (cluster of differentiation) 14 is a cell-surface glycoprotein expressed in macrophages, monocytes, dendritic cells, and neutrophils [28]. CD14 is a lipopolysaccharide (LPS) receptor. It transfer the LPS signal from bacteria via Toll-like receptor-4 [29], triggers the release of proinflammatory cytokines, and activates a systematic inflammatory response [30]. Presepsin consists of the N-terminal 13 kDa fragment of the CD14 protein [31]. Several recent studies presented presepsin as a promising diagnostic biomarker for adult sepsis [32–35]. A current systematic review reported that presepsin had high sensitivity and specificity in predicting neonatal sepsis [36]. Presepsin levels increase within 2 h and peak at 3 h after the onset of infection [24]. Serum presepsin can be measured easily and rapidly [37]. Therefore, presepsin could be a useful biomarker for the early diagnosis of sepsis. Several clinical studies [38–41] proposed the diagnostic value of presepsin in pediatric sepsis. However, there is no published meta-analysis of its diagnostic efficacy in pediatric sepsis patients.

Therefore, we conducted a systematic review and meta-analysis to assess the diagnostic value of presepsin in pediatric sepsis and compare it with other biomarkers.

## Methods

This meta-analysis was conducted and reported in compliance with the Preferred Reporting Items for Systematic Reviews and Meta-Analyses Statement (PRISMA) [42].

### Search strategy

Medline/Pubmed, Embase, the Cochrane Library, and ISI Web of Science were searched for studies reporting the performance of presepsin in the diagnosis of pediatric sepsis. The search algorithms used for each database are shown in Additional file 1. The search was executed on February 13, 2019. Reference lists were also screened for pertinent articles and all languages were included in the search.

### Study selection

Two reviewers (SHY) and (JGY) independently evaluated the eligibility of the studies. In cases of disagreement, a third reviewer (HYK) was also consulted. No date restrictions were applied to the study search. Studies included were those reporting on the efficacy of serum presepsin in the diagnosis of pediatric sepsis. The pediatric age range was defined as > 4 wks weeks and <  18 y. All article titles and abstracts were screened for relevance and eligibility.

Studies evaluating the accuracy of presepsin in the diagnosis of pediatric sepsis and those providing sufficient data to extract a 2 × 2 contingency table were included. The presence of infection was defined as microbiologically confirmed (smear microscopy, culture method, or PCR) or suspected as probable according to a clinical record review [11]. Studies addressing catheter-related bloodstream infections (CRBSI) were eligible as CRSBI is a bacteremia originating from an intravascular device [43, 44]. Full texts were retrieved for all articles meeting the aforementioned criteria.

Articles were excluded if they did not address sepsis or assess presepsin. Studies that did not separately consider pediatric patients were also omitted. Reviews, letters, editorials, expert opinions, and animal experiments were also ruled out. Finally, reports with overlapping data or with insufficient data to calculate presepsin sensitivity or specificity were eliminated.

### Data extraction

The following variables were independently extracted by two authors (SHY and JGA): first author, publication year, country, type of study, clinical setting, age range at diagnosis, sample size, number of patients and controls, sample type, cutoff value, inclusion and exclusion criteria, diagnostic references, and presepsin assay methods. Authors were contacted by e-mail and additional data were requested in the event of information gaps. For studies consisting of multiple groups and/or different backgrounds, the analysis of each group was treated as a single study.

### Quality assessment

The methodological quality of each study was tested with the Quality Assessment Tool for Diagnostic Accuracy Studies (QUADAS-2 score) [45]. This evaluation includes the following bias risk assessment domains: patient selection, index test, reference standard, flow and timing, sources of variation (applicability), and reporting quality.

### Statistical analysis

Accuracy data (true positive, false positive, false negative, and true negative) were extracted for each study. A 2 × 2 contingency table was constructed to calculate sensitivity, specificity, positive and negative likelihood ratios (LR), and diagnostic odds ratio (DOR) with their corresponding 95% confidence intervals (95% CI). A value of 0.5 was added to all cells for studies with zero values to correct for continuity. Results were evaluated by forest plots. Heterogeneity of sensitivity and specificity was evaluated by a χ^2^ test. *P* < 0.10 indicated significant heterogeneity. In this case, a bivariate random effects model was adopted [46, 47].

The threshold effect is a major source of heterogeneity in meta-analyses of diagnostic tests [48]. The studies included used different cutoff values for presepsin in sepsis diagnosis. Therefore, a threshold effect is anticipated. Therefore, Spearman’s correlation coefficient (r) was calculated. R ≥ 0.6 indicated a threshold effect [49].

Summary sensitivity and specificity were calculated with a bivariate model and a hierarchical summary receiver operating characteristic (HSROC) model [48].

R v. 3.5.2 (http://www.R-project.org) was used for the statistical and meta-analyses. Publication bias was measured with funnel plots. Egger’s test evaluated funnel plot asymmetry [50]. The trim-and-fill method corrected for any funnel plot asymmetry resulting from publication bias [51].

## Results

**Fig. 1:**
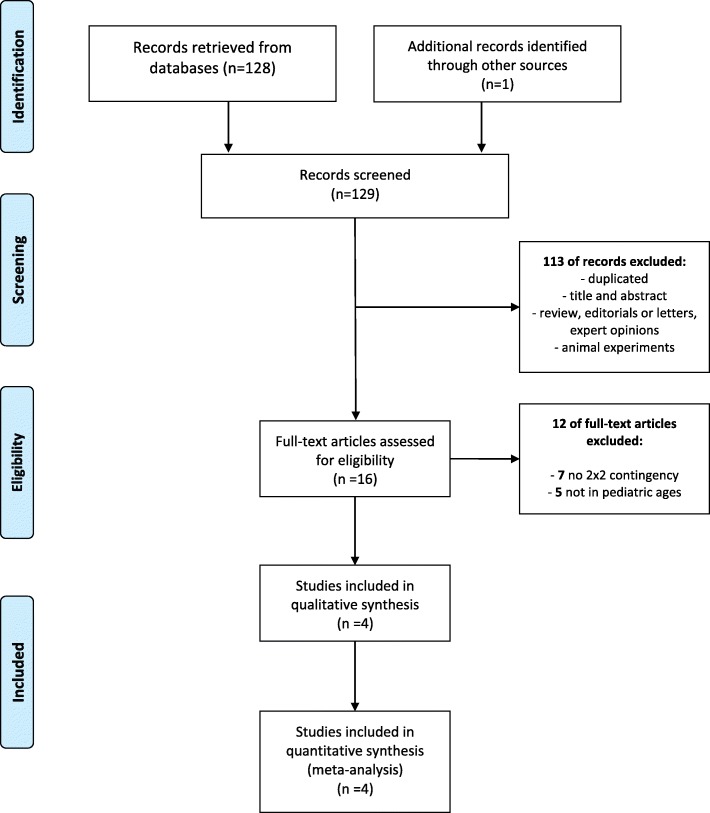
Flow diagram of the study selection process

One hundred and twenty-nine published studies were gleaned from our electronic database search. Of these, 113 were excluded after title and abstract screening and the remaining 15 were subjected to full text reviews (Fig. 1). Of these, seven had insufficient data to construct a 2 × 2 contingency table and five did not include children or adolescents.

Three studies met our criteria. Of these, one presented two sets of results from different patients/controls. Thus, it was included as a separate study. Thus, four studies were included in our qualitative assessment and meta-analysis [38–40] (Fig. 1). The characteristics of these studies are listed in Table 1.

**Table 1:** Summary of the included studies

No	Author (Year)	Country	Type of study	Age (range)	Sample Size (all)	Patients/control (*n*)	Cutoff (ng mL^-l^)	Sample	Inclusion criteria	Exclusion	References	Assay
1	Tanır Basaranoglu 2018	Turkey	Prospective	1mo–18 y	138	58 sepsis/80 (healthy controls)	990	serum	1 mo–18 y, clinical signs of CRBSI	received antibiotics within the 24 h of presentation with fever	2009 IDSA guideline	ELISA
2	Baraka 2018	Egypt	Case-control	2–15 y	60	18 sepsis/42 (non-sepsis patients)	1014	plasma	< 16 y, pediatric patients with HM, during episodes of fever and neutropenia after receiving CTx	age > 16 y, non-HM pediatric patients, not on chemotherapy	blood culture	PATHFAST
3	Plesko 2016–1	Slovakia	Prospective	1.5–18.9 y	55	12 sepsis/43 (non-sepsis patients)	240	unspecified	< 18.9 y, pediatric patients with HM, the presence of fever, hypothermia, chills, or another sign of possible sepsis	Febrile episodes thought to be an adverse effect of CTx, not having blood culture drawn, patients with proven non-bacterial infection	blood culture	PATHFAST
3	Plesko 2016–2	Slovakia	Prospective	1.5–18.9 y	55	13 sepsis/42 (non-sepsis patients)	299	unspecified	< 18.9 y, pediatric patients with HM, the presence of fever, hypothermia, chills, or another sign of possible sepsis	Febrile episodes thought to be an adverse effect of CTx, not having blood culture drawn, patients with proven non-bacterial infection	modified IPSCC definition (2005)	PATHFAST

*CRBSI* catheter-related blood stream infection, *CTx* chemotherapy, *HM* hematologic malignancy, *IDSA* Infectious Diseases Society of America, *IPSCC* International Pediatric Sepsis Consensus Conference

All eligible studies were published between 2016 and 2018. One was conducted in Turkey [38], one in Egypt [39], and two in Slovakia [40]. Three of them were prospective studies and one was retrospective. Three hundred and eight patients were included in the four studies. Of the 207 (67.2%) patients in the control group, 127 (61.4%) were categorized as non-sepsis patients and 80 (38.6%) as healthy volunteers. There were 101 patients with sepsis (32.8%).

Patient populations were heterogeneous (Table 1). Three studies included patients with hematological malignancy (HM), and one included patients with CRBSI. Of the three studies with HM patients, one included those on chemotherapy with febrile neutropenia [39] and the other two included those presenting with fever, hypothermia, chills, or other putative signs of sepsis [40].

Two studies identified sepsis patients by positive bacterial cultures [39, 40]. One study [38] defined sepsis patients according to the criteria of the Infectious Diseases Society of America (IDSA) consensus conference (2009) [44]. Another study [40] designated sepsis patients based on the modified International Pediatric Sepsis Consensus Conference definition (IPSCC) (2005) [7].

One study used serum [38], one used plasma [39], and the other used unspecified blood samples [40] to test for sepsis. Three studies used PATHFAST [39, 40], and another used ELISA [38] to assay for presepsin. The presepsin cutoff values for sepsis detection ranged from 240 to 1014 pg mL^-l^.

### Quality assessment

**Fig. 2:**
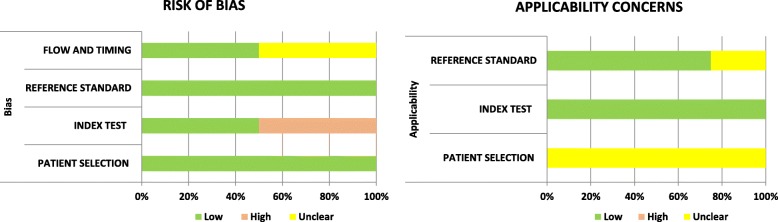
Quality Assessment of the Diagnostic Accuracy Studies-2 (QUADAS-2) criteria for the included studies

The output of the QUADAS-2 tool is summarized in Fig. 2. Two studies evaluated the optimal cutoff value to calculate sensitivity and specificity instead of using the predefined value scored ‘high risk’ in the index test domain. In terms of the patient selection domain, the risk of bias was low in most studies as they clearly defined their exclusion criteria. Two studies did not include all patients in their analyses and were scored ‘unclear’ in the patient flow and timing domains. All studies had low risk in the reference standard domain as they used positive blood culture, the guidelines of the IDSA consensus conference [44], and the IPSCC definition [7] to diagnose sepsis.

Most of the studies scored ‘unclear’ in terms of the patient selection domain as the majority of the enrolled patients had HM. One study scored ‘unclear’ in the reference standard domain as it defined the disease condition as CRBSI which limited its applicability.

### Meta-analysis for diagnostic accuracy

**Fig. 3:**
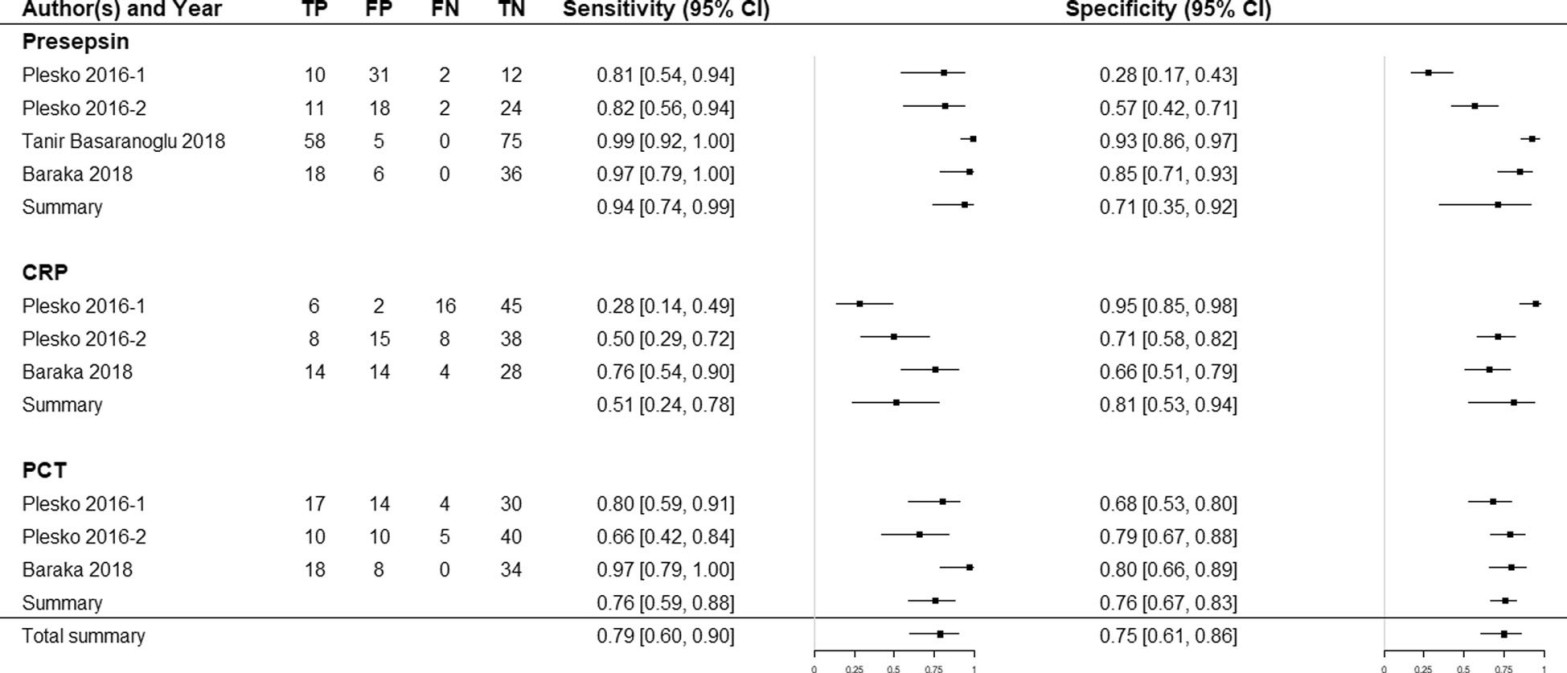
Forest plots of the sensitivity and specificity of presepsin, C-reactive protein (CRP), and procacitonin (PCT) for the diagnosis of pediatric sepsis

Descriptive statistics of the diagnostic accuracy of presepsin are presented in the form of a forest plot in Fig. 3. Significant heterogeneity between studies was noted in terms of sensitivity (*χ*^2^ = 11.17; *P* = 0.0108) and specificity (*χ*^2^ = 65.78; *P* < 0.0001). Spearman’s correlation coefficient (r) was − 0.938 (95% CI; − 0.999 to 0.234). Thus, there was no threshold effect (Additional file 2).

**Fig. 4:**
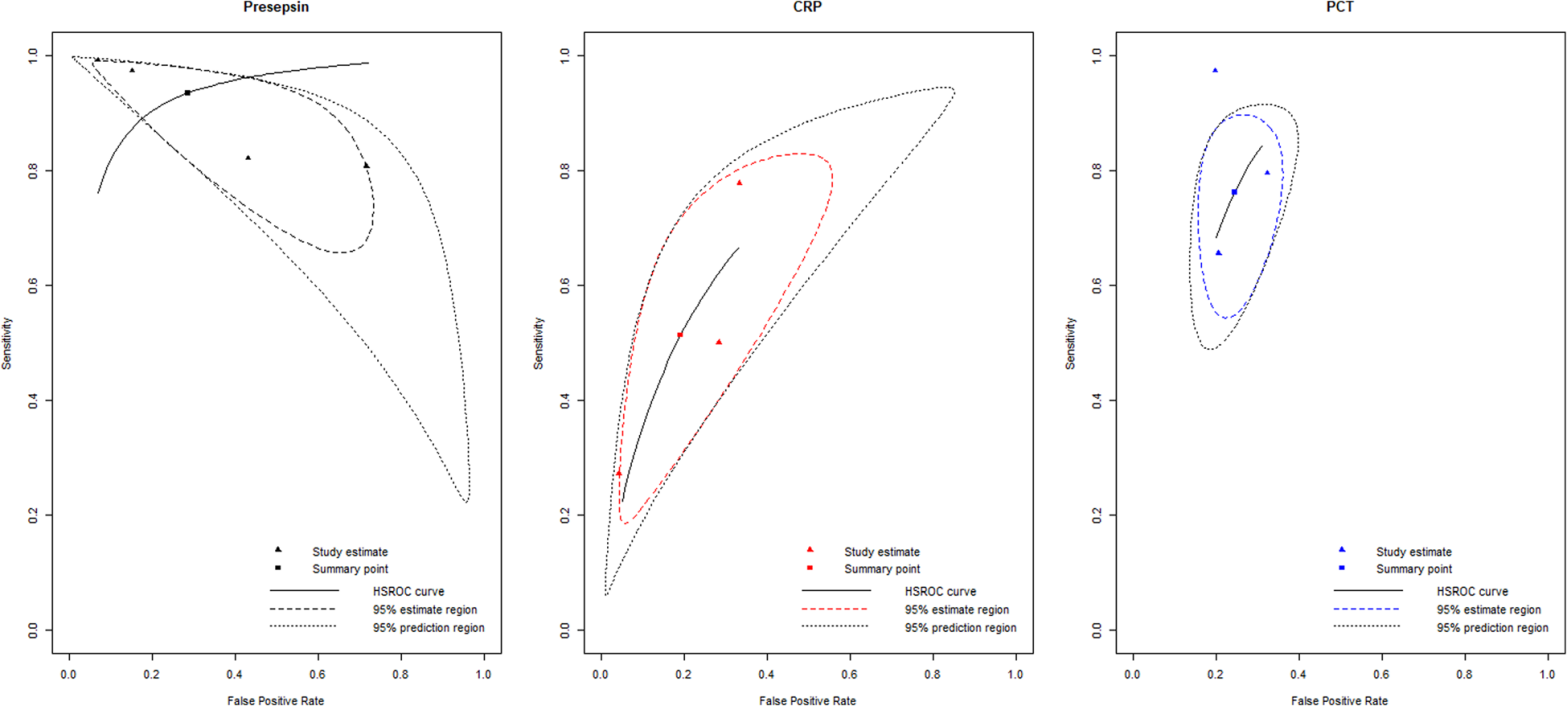
Hierarchical summary receiver operating characteristic (HSROC) curve of the diagnostic accuracy of presepsin, C-reactive protein (CRP), and procalcitonin (PCT) for pediatric sepsis. Summary points of the sensitivity and specificity, HSROC curve, 95% confidence region, and 95% prediction region are provided. The area under the curve of the HSROC for presepsin, CRP, and PCT were 0.925, 0.715, and 0.820, respectively

Summary estimates of sensitivity, specificity, positive LR, negative LR, and DOR were calculated with a bivariate random effects model (Table 2). The overall sensitivity was 0.94 (95% CI; 0.74–0.99) and the specificity was 0.71 (95% CI; 0.35–0.92). The area under the [HSROC] curve (AUC) was 0.925 (Fig. 4). Therefore, presepsin had a high diagnostic accuracy for pediatric sepsis.

**Table 2:** Summary estimates of the diagnostic accuracy of presepsin, C-reactive protein, procalcitonin to diagnose pediatric sepsis

Author(s) and year	Sensitivity (95% CI)	Specificity (95% CI)	Positive LR (95% CI)	Negative LR (95% CI)	DOR (95% CI)
Presepsin					
Plesko 2016–1	0.81 [0.54, 0.94]	0.28 [0.17, 0.43]	1.13 [0.82, 1.56]	0.68 [0.20, 2.27]	1.67 [0.36, 7.67]
Plesko 2016–2	0.82 [0.56, 0.94]	0.57 [0.42, 0.71]	1.91 [1.25, 2.91]	0.31 [0.10, 0.99]	6.09 [1.37, 27.17]
Tanir Basaranoglu 2017	0.99 [0.92, 1.00]	0.93 [0.86, 0.97]	14.60 [6.51, 32.73]	0.01 [0.001, 0.14]	1606.09 [87.05, 29,633.96]
Baraka 2018	0.97 [0.79, 1.00]	0.85 [0.71, 0.93]	6.44 [3.16, 13.13]	0.03 [0.002, 0.48]	207.77 [11.09, 3892.29]
Bivariate summary estimates (95% CI)	0.94 [0.74, 0.99]	0.71 [0.35, 0.92]	3.24 [1.14, 12.38]	0.08 [0.01, 0.74]	32.87 [2.12, 510.09]
CRP					
Plesko 2016–1	0.28 [0.14, 0.49]	0.95 [0.85, 0.98]	5.43 [1.38, 21.38]	0.76 [0.58, 0.99]	7.17 [1.50, 34.24]
Plesko 2016–2	0.50 [0.29, 0.72]	0.71 [0.58, 0.82]	1.74 [0.92, 3.29]	0.70 [0.42, 1.16]	2.48 [0.81, 7.60]
Baraka 2018	0.76 [0.54, 0.90]	0.66 [0.51, 0.79]	2.26 [1.39, 3.69]	0.36 [0.16, 0.82]	6.33 [1.85, 21.72]
Bivariate summary estimates (95% CI)	0.51 [0.24, 0.78]	0.81 [0.53, 0.94]	2.68 [0.51, 13.00]	0.60 [0.23, 1.43]	4.63 [2.16, 9.95]
PCT					
Plesko 2016–1	0.80 [0.59, 0.91]	0.68 [0.53, 0.80]	2.47 [1.54, 3.97]	0.30 [0.13, 0.71]	8.18 [2.44, 27.43]
Plesko 2016–2	0.66 [0.42, 0.84]	0.79 [0.67, 0.88]	3.19 [1.67, 6.08]	0.43 [0.22, 0.86]	7.36 [2.14, 25.32]
Baraka 2018	0.97 [0.79, 1.00]	0.80 [0.66, 0.89]	4.93 [2.69, 9.04]	0.03 [0.002, 0.51]	150.18 [8.20, 2750.01]
Bivariate summary estimates (95% CI)	0.76 [0.59, 0.88]	0.76 [0.67, 0.83]	3.17 [1.79, 5.18]	0.32 [0.61, 0.14]	11.88 [3.49, 40.47]
Bivariate summary estimates (95% CI) (Total)	0.79 [0.60, 0.90]	0.75 [0.61, 0.86]	3.16 [1.54, 6.43]	0.28 [0.66, 0.12]	10.76 [4.31, 26.83]

*CRP* C-reactive protein, *PCT* procalcitonin, *CI* confidence interval, *LR* likelihood ratio, *DOR* diagnostic odds ratio

**Fig. 5:**
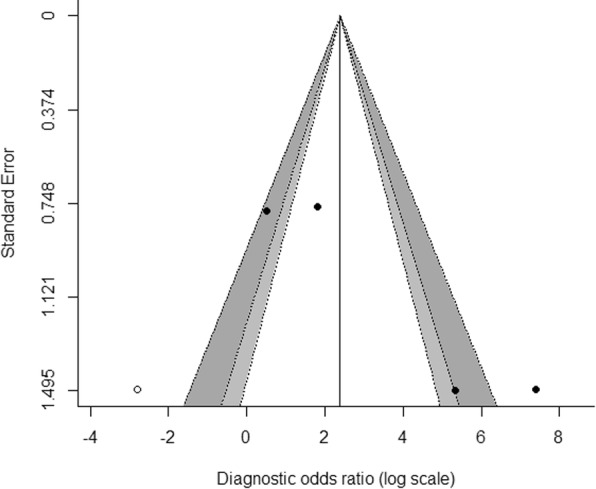
Trim-and-fill funnel plot of the log of diagnostic odds ratio (DOR) of presepsin to diagnose pediatric sepsis. The x-axis represents the study result [log (DOR)]. The y-axis represents the study precision [the standard error of log (DOR)]. Each filled dot represents one study

An asymmetric funnel plot and Egger’s test (*P* = 0.0001) revealed publication bias among the studies (Additional file 3). The trim-and-fill method was applied for bias correction and showed log DOR = 2.39 for presepsin corresponding to a pooled DOR = 10.91 (Fig. 5).

The presepsin cutoff value for detecting pediatric sepsis varies, ranging from 240 to 1014 pg/ml. Mean cutoff value was 635.8 pg/ml. Due to the varied cutoff values between studies, we conducted subgroup analysis using a cutoff value of 650 pg/ml. Two studies [38, 39] featured cutoff values higher than 650 pg/ml and the other two studies [40] had cutoff values lower than 650 pg/ml. We found higher pooled sensitivity (0.84 vs. 0.99) and specificity (0.42 vs. 0.90) in the studies where cutoff value was greater than 650 pg/ml. This cutoff value featured higher diagnostic accuracy as well (AUC 0.827 vs. 0.983) (Table 3, Additional file 4).

**Table 3:** Diagnostic accuracy of presepsin according to the cut off value

	Number of studies	Sensitivity (95% CI)	Specificity (95% CI)	Positive LR (95% CI)	Negative LR (95% CI)	DOR (95% CI)	AUC
Cutoff ≤650 pg/ml	2	0.84 [0.64, 0.94]	0.42 [0.18, 0.71]	1.45 [0.78, 3.24]	0.38 [0.08, 2.00]	3.81 [1.03, 14.04]	0.827
Cutoff > 650 pg/ml	2	0.99 [0.88, 1.00]	0.90 [0.78, 0.96]	9.9 [4.00, 25.00]	0.01 [0.001, 0.15]	580.75 [73.53, 4586.94]	0.983

*CI* confidence interval, *LR* likelihood ratio, *DOR* diagnostic odds ratio, *AUC* area under the receiver operating characteristic curve

### Comparison of the performances of presepsin, CRP, and PCT

Three studies compared presepsin with CRP and PCT. Two of these defined the patient by positive blood culture [38, 40] and one used the modified IPSCC definition [40]. Descriptive statistics of the diagnostic accuracy of CRP and PCT for sepsis detection are presented in a forest plot (Fig. 3) and summarized in Table 2.

The pooled sensitivity of presepsin was higher than those of CRP and PCT [presepsin: 0.94 (95% CI: 0.74–0.99); CRP: 0.51 (95% CI: 0.24–0.78); PCT: 0.76 (95% CI: 0.59–0.88)]. In contrast, the pooled specificity of presepsin was lower than that of CRP and PCT [presepsin: 0.71 (95% CI: 0.35–0.92); CRP: 0.81 (95% CI: 0.53–0.94); PCT: 0.76 (95% CI: 0.67–0.83)]. The AUC of presepsin (0.925) was higher than that of CRP (0.715) and PCT (0.830) (Fig. 4) Thus, presepsin had a higher diagnostic performance than CRP or PCT. Threshold effects could not be calculated for CRT and PCT as there were too few studies involving them. Funnel plots of the studies including CRP and PCT indicated no publication bias (Additional file 3).

## Discussion

The present study summarizes the overall performance of presepsin in the diagnosis of pediatric sepsis and is based on currently available literature. To the best of our knowledge, this is the first systematic review and meta-analysis to evaluate the diagnostic value of presepsin for pediatric sepsis (not including neonates). Presepsin had high sensitivity and moderate specificity. Presepsin also displayed high diagnostic performance in predicting pediatric sepsis. Thus, presepsin may be a useful diagnostic biomarker for pediatric sepsis.

To date, several meta-analyses of the diagnostic accuracy of presepsin in adult sepsis have been published. They reported pooled sensitivities ranging from 0.78–0.86, pooled specificities ranging from 0.73–0.83, positive likelihood ratios ranging from 3.40–4.63, negative likelihood ratios ranging from 0.18–0.22, DOR ranging from 14.25–22.0, and AUC ranging from 0.86–0.89 [32–35, 52, 53]. Two recent meta-analyses indicated pooled presepsin sensitivities and specificities each in the range 0.9–0.91 for the diagnosis of neonatal sepsis [36, 54]. The DOR was in the range 120.94–170.28 and the AUC was in the range 0.968–0.975 [36, 54]. The present study showed higher sensitivity and similar specificity of presepsin for the diagnosis of pediatric sepsis relative to those for adult sepsis. For the diagnosis of pediatric sepsis, however, presepsin had similar sensitivity and lower specificity compared to neonatal sepsis analysis. As it has high sensitivity, presepsin may be very useful for the exclusion of sepsis in pediatric patients when the level of this biomarker is normal or lower than the cutoff value. Nevertheless, the specificity of presepsin is only moderate. Consequently, it may be necessary to diagnose sepsis by correlating it with clinical symptoms as false positives could occur.

### Presepsin level according to the etiologies of sepsis

Acute-phase reactants, such as CRP, PCT, and presepsin are a class of serum proteins that change in response to inflammation from infections, surgery, trauma, autoimmune disorders, and malignancy [15, 55, 56]. The level of acute phase reactants usually reflects the presence and degree of an inflammatory process so they are thought to have the potential capacities in early diagnosis by differentiating sepsis from non-infectious systemic inflammation, as prognostic implications, and in antibiotic guidance strategies [15, 57–59]. However, it is believed that the level of acute phase reactant is not specific to any particular disease, nor can they precisely distinguish infection from other etiologies of acute and chronic inflammation [56, 60, 61]. Furthermore, there are still not enough studies to show whether the level of acute phase reactants would change according to the specific microorganism [15, 61, 62].

In our review, two studies reported the level of presepin according to the causing organisms. One study [38] showed that median presepsin values were similar within the groups of microorganisms; the median presepsin value was 1668 pg/ml (range: 1048–2935) for gram-positive bacteremia patients, 1756 pg/ml (range: 1103–2033) for gram-negative bacteremia patients, and 1406 pg/ml (range: 1348–1566) for candidemia patients.

The other study [40] reported that the mean log value of presepsin was 2.47 (SD 0.13) (pg/ml) for gram positive bacteremia patients and 2.64 (SD 0.29) (pg/ml) for gram negative bacteremia patients with no statistical difference (*P* = 0.237). However, we could not conduct subgroup analysis due to insufficient data.

### Cut off values of presepsin for diagnosing sepsis

Cutoff value of presepsin for diagnosing sepsis varies according to the different reference tests, controls, and clinical settings. In recent adult meta-analysis, cutoff values ranged from 317 to 849 pg/ml and the optimal cut off value was 600–650 pg/ml [35]. The authors found similar sensitivity (0.85 vs. 0.84) but lower pooled specificity in the cutoff values greater than 700 pg/ml studies, compared to values smaller than 700 pg/ml studies (0.59 vs. 0.80) [35]. In recent neonatal meta-analysis, presepsin cutoff value ranged from 304.5 to 885.0 pg/ml and when using a cutoff value < 600 ng/L, sensitivity was 0.93, with a specificity of 0.81 and AUC 0.8195; using a cut-off value of > 600 ng/L resulted in sensitivity of 0.87 and specificity of 0.97, with higher diagnostic accuracy (AUC 0.976) [54]. However, the other neonatal meta-analysis reported that its diagnostic efficacy was maximized (sensitivity 0.91, specificity 0.97, AUC 0.99) when using a presepsin cutoff value ranging from 650 to 850 pg/ml [36].

In our study, the presepsin cutoff value for detecting pediatric sepsis varies, ranging from 240 to 1014 pg/ml. We found higher pooled sensitivity, specificity and diagnostic accuracy in the cutoff value greater than 650 pg/ml. However, a small sample size and heterogeneous characteristics of included studies are limitations disallowing the application of these results from clinical practice at this stage.

### Comparison of CRP, and PCT as diagnostic biomarkers for sepsis

CRP and PCT are the most commonly used diagnostic biomarker for pediatric sepsis and their diagnostic accuracies have been extensively studied [16, 25]. Several clinical studies showed that PCT has higher diagnostic accuracy than CRP in the differentiation of sepsis and SIRS (PCT: AUC = 0.71–0.99; CRP: AUC = 0.54–0.65) [63, 64]. A recent meta-analysis investigated the diagnostic accuracy of PCT in the distinction of sepsis from SIRS. PCT had good sensitivity (pooled, 0.78) and poor specificity (pooled, 0.57) at a cutoff value of < 2.0 ng mL^-l^ in pediatric patients older than neonates [11].

Several head-to-head comparisons were made between presepsin and PCT or CRP in adult patients. A recent adult patient meta-analysis disclosed no statistically significant difference in the diagnostic accuracy of sepsis between presepsin and CRP (presepsin: AUC = 0.85; CRP: AUC = 0.85) or between presepsin and PCT (presepsin: AUC = 0.87; PCT: AUC = 0.86) [35]. For studies conducted on patients in intensive care units, the pooled sensitivity of presepsin was higher than that of PCT (0.88 vs. 0.75), but lower than that of PCT (0.58 vs. 0.75) [35]. Another meta-analysis compared the diagnostic value of PCT with that of presepsin for sepsis in critically ill adult patients. The pooled sensitivities (0.84 for presepsin and 0.8 for PCT) and specificities (0.73 for presepsin and 0.75 for PCT) did not significantly differ between the two biomarkers [65].

Presepsin may be the most accurate biomarker of neonatal sepsis. One study showed that presepsin had higher AUC than CRP (0.975 vs. 0.858) and PCT (0.959 vs. 0.783) [36]. In the present study, the overall sensitivity of presepsin was higher than that of CRP (0.94 vs. 0.51) and PCT (0.94 vs. 0.76). However, the overall specificity of presepsin was lower than that of CRP (0.71 vs. 0.81) and PCT (0.71 vs. 0.76). Moreover, the AUC of presepsin was higher than that of CRP (0.925 vs. 0.715) and PCT (0.925 vs. 0.830). These results suggest that the overall diagnostic performance of presepsin is greater than that of CRP and PCT. Furthermore, presepsin levels increase earlier in response to sepsis than either CRP or PCT [24]. Thus, presepsin may be promising as a sensitive biomarker for the early diagnosis of sepsis in pediatric patients.

To the best of our knowledge, this systematic review and meta-analysis is the first to evaluate the performance of presepsin as a diagnostic biomarker of sepsis in pediatric patients (excluding neonates) and to compare it with PCT and CRP. Nevertheless, our study had several limitations. First, only four publications were included as there was relatively little currently available data for pediatric sepsis patients. Second, differences in the reference standards, control group definitions, and specimen types in the included studies are possible sources of heterogeneity. Moreover, we could not conduct a subgroup analysis as the number of included studies was very small. Third, the included studies comprised mainly enrolled HM pediatric patients. Three of four studies included in the meta-analysis were conducted in pediatric patients with hematologic malignancies. Thus, this is a major limitation of the study, and the conclusions cannot be generalized for all pediatric populations. Therefore, a more prospective clinical study with a larger sample size is necessary to compensate for the aforementioned deficiencies.

## Conclusions

Presepsin showed higher sensitivity and accuracy but relatively lower specificity for the diagnosis of pediatric sepsis than either PCT or CRP.

Owing to the small number and heterogeneity of the included studies, however, the foregoing results should be carefully interpreted and applied. Future clinical trials are required to validate and determine the optimal presepsin cutoff for the diagnosis of sepsis in children.

## Additional files

Additional file 1: Search Strategy. (DOCX 16 kb)

Additional file 2: Inter-study heterogeneity and threshold effect. (DOCX 19 kb)

Additional file 3: Funnel plots for the assessment of publication bias. (DOCX 127 kb)

Additional file 4: Inter-study heterogeneity (subgroup analysis according to the cut-off value). (DOCX 19 kb)

## Data Availability

The data used in the present study are appropriately cited.

## Abbreviations

AUC: Area under the receiver operating characteristic curve
CD: Cluster of differentiation
CI: Confidence interval
CRP: C-reactive protein
DOR: Diagnostic odds ratio
HM: Hematological malignancy
HSROC: Hierarchical summary receiver operating characteristic
IDSA: Infectious Diseases Society of America
LPS: Lipopolysaccharide
LR: Likelihood ratio
PCT: Procalcitonin
PLR: Positive likelihood ratio
PRISMA: Preferred Reporting Items for Systematic Reviews and Meta-Analyses Statement
QUADAS: Quality Assessment of Diagnostic Accuracy Studies
SIRS: Systemic inflammatory response syndrome
