# Supplementary material for: Presepsin as a diagnostic marker of sepsis in children and adolescents: a systemic review and meta-analysis
Source: BMC Infect Dis. 2019 Aug 30;19:760. doi: 10.1186/s12879-019-4397-1 (PMC6717384; doi:10.1186/s12879-019-4397-1)
Supplement: Supplementary file 1 — Search Strategy. (DOCX 16 kb) [file 12879_2019_4397_MOESM1_ESM.docx]

**Additional file 1. Search Strategy**

**< Medline/Pubmed >**

| Search | Query |
| --- | --- |
| #5 | Search (((((Bacteremia[Mesh] OR Sepsis[Mesh] OR Shock, Septic[Mesh] OR bacteremi*[TIAB] OR bacteraemi*[TIAB] OR septic*[TIAB]))) AND  ((presepsin[TIAB] OR presepsin protein, human[NM] OR soluble CD14[TIAB] OR sCD14[TIAB]))) AND ((Child[MeSH] OR infant[Mesh]  OR Adolescent[Mesh] OR child*[TIAB] OR "infant*"[TIAB] OR adolescent*[TIAB] OR p*ediatric [TIAB]))) NOT ((Infant, Newborn [MESH] OR neonat*[TIAB])) |
| #4 | Search (Infant, Newborn [MESH] OR neonat*[TIAB]) |
| #3 | Search (Child[MeSH] OR infant[Mesh] OR Adolescent[Mesh] OR child*[TIAB] OR "infant*"[TIAB] OR adolescent*[TIAB] OR p*ediatric [TIAB]) |
| #2 | Search (presepsin[TIAB] OR presepsin protein, human[NM] OR soluble CD14[TIAB] OR sCD14[TIAB]) |
| #1 | Search (Bacteremia[Mesh] OR Sepsis[Mesh] OR Shock, Septic[Mesh] OR bacteremi*[TIAB] OR bacteraemi*[TIAB] OR septic*[TIAB]) |

**< Cochrane Library >**

ID Search

#1 MeSH descriptor: [Bacteremia] explode all trees

#2 MeSH descriptor: [Shock, Septic] explode all trees

#3 MeSH descriptor: [Sepsis] explode all trees

#4 (bacter*emi*):ti,ab,kw

#5 (sepsis):ti,ab,kw

#6 (septic*):ti,ab,kw

#7 ("septic shock"):ti,ab,kw

#8 #1 OR #2 OR #3 OR #4 OR #5 OR #6 OR #7

#9 MeSH descriptor: [Child] explode all trees

#10 MeSH descriptor: [Infant] explode all trees

#11 MeSH descriptor: [Adolescent] explode all trees

#12 ("p*ediatric"):ti,ab,kw

#13 (child*):ti,ab,kw

#14 (adolescent*):ti,ab,kw

#15 (infant*):ti,ab,kw

#16 MeSH descriptor: [Infant, Newborn] explode all trees

#17 (neonat*):ti,ab,kw

#18 #9 OR #10 OR #11 OR #12 OR #13 OR #14 OR #15

#19 #16 OR #17

#20 (sCD14):ti,ab,kw

#21 (sCD 14):ti,ab,kw

#22 (presepsin):ti,ab,kw

#23 (soluble cd 14):ti,ab,kw

#24 (soluble cd14):ti,ab,kw

#25 #20 OR #21 OR #22 OR #23 OR #24

#26 #8 AND #18 AND #25

#27 #26 NOT #19

**< ISI Web of Science >**

| ID | Search |
| --- | --- |
| # 14 | #13 AND #12 AND #11 |
| # 13 | #10 OR #9 OR #8 OR #7 |
| # 12 | #6 OR #5 OR #4 OR #3 OR #2 OR #1 |
| # 11 | ALL=((child* OR "infant*" OR adolescent* OR p*ediatric) NOT ("neonat*" OR "newborn*")) |
| # 10 | ALL= (sCD14) |
| # 9 | ALL= (“soluble CD14”) |
| # 8 | ALL= ("presepsin protein, human") |
| # 7 | ALL=(presepsin) |
| # 6 | ALL=("septic shock") |
| # 5 | ALL=(septic*) |
| # 4 | ALL=(Sepsis) |
| # 3 | ALL=(bacteraemi*) |
| # 2 | ALL=(bacteremi*) |
| # 1 | ALL=(Bacteremia) |

**< Embase >**

No. Query

#82. #81 AND ([adolescent]/lim OR [child]/lim OR [infant]/lim OR [preschool]/lim OR [school]/lim)

#81. #79 AND #80

#80. #72 OR #75 OR #78

#79. #60 OR #63 OR #66 OR #69

#78. #76 OR #77

#77. 'scd14':ti,ab

#76. 'scd14'

#75. #73 OR #74

#74. 'soluble cd14':ti,ab

#73. 'soluble cd14'

#72. #70 OR #71

#71. 'presepsin':ti,ab

#70. 'presepsin'/exp

#69. #67 OR #68

#68. 'septic shock':ti,ab

#67. 'septic shock'/exp

#66. #64 OR #65

#65. 'septic*':ti,ab

#64. 'septicemia'/exp

#63. #61 OR #62

#62. ‘bacter$emi*’:ti,ab

#61. 'bacteremia'/exp

#60. #58 OR #59

#59. 'sepsis':ti,ab

#58. 'sepsis'/exp

#57. #55 AND #56

#56. #48 OR #51 OR #54

#55. #36 OR #39 OR #42 OR #45

#54. #52 OR #53

#53. 'scd14':ti,ab

#52. 'scd14'

#51. #49 OR #50

#50. 'soluble cd14':ti,ab

#49. 'soluble cd14'

#48. #46 OR #47

#47. 'presepsin':ti,ab

#46. 'presepsin'/exp

#45. #43 OR #44

#44. 'septic shock':ti,ab

#43. 'septic shock'/exp

#42. #40 OR #41

#41. 'septic*':ti,ab

#40. 'septicemia'/exp

#39. #37 OR #38

#38. ‘bacter$emi*’:ti,ab

#37. 'bacteremia'/exp

#36. #34 OR #35

#35. 'sepsis':ti,ab

#34. 'sepsis'/exp

#33. #31 OR #32

#32. 'scd14':ti,ab

#31. 'scd14'

#30. #28 OR #29

#29. 'soluble cd14':ti,ab

#28. 'soluble cd14'

#27. #25 OR #26

#26. 'presepsin':ti,ab

#25. 'presepsin'/exp

#24. #22 OR #23

#23. 'septic shock':ti,ab

#22. 'septic shock'/exp

#21. #19 OR #20

#20. 'septic*':ti,ab

#19. 'septicemia'/exp

#18. #16 OR #17

#17. ‘bacter$emi*’:ti,ab

#16. 'bacteremia'/exp

#15. #13 OR #14

#14. 'sepsis':ti,ab

#13. 'sepsis'/exp

#12. #10 OR #11

#11. 'septic shock':ti,ab

#10. 'septic shock'/exp

#9. #7 OR #8

#8. 'septic*':ti,ab

#7. 'septicemia'/exp

#6. #4 OR #5

#5. ‘bacter$emi*’:ti,ab

#4. 'bacteremia'/exp

#3. #1 OR #2

#2. 'sepsis':ti,ab

#1. 'sepsis'/exp
