# Supplementary material for: Presepsin as a diagnostic marker of sepsis in children and adolescents: a systemic review and meta-analysis
Source: BMC Infect Dis. 2019 Aug 30;19:760. doi: 10.1186/s12879-019-4397-1 (PMC6717384; doi:10.1186/s12879-019-4397-1)
Supplement: Supplementary file 2 — Inter-study heterogeneity and threshold effect. (DOCX 19 kb) [file 12879_2019_4397_MOESM2_ESM.docx]

**Additional File 2. Inter-study heterogeneity and threshold effect.**

|  | Heterogeneity | | Threshold effect | AUC |
| --- | --- | --- | --- | --- |
|  | Sensitivity | Specificity |  |  |
| Presepsin | $\chi^{2}=11.17,$*P* = 0.0108 | $\chi^{2}=65.78,$*P* < 0.0001 | -0.938 (-0.999, 0.234) | 0.925 |
| CRT | $\chi^{2}=10.10$, *P* = 0.0064^,^ | $\chi^{2}=13.06$, *P* = 0.0015 | - | 0.715 |
| PCT | $\chi^{2}=5.92$, *P* = 0.0518 | $\chi^{2}=2.41$, *P* = 0.3000 | - | 0.820 |
| Total | $\chi^{2}=68.84$, *P* < 0.0001 | $\chi^{2}=87.37$, *P* < 0.0001 | 0.131 (-0.543, 0.703) | 0.833 |

CRP, C-reactive protein; PCT, procalcitonin; AUC, area under the curve. Heterogeneity of the sensitivity and specificity variables was evaluated with a χ^2^ test. *P* < 0.10 for χ^2^ test indicates substantial heterogeneity. Threshold effect calculated using Spearman’s correlation coefficient, r. Threshold effect when r ≥ 0.6.
