# Supplementary material for: Presepsin as a diagnostic marker of sepsis in children and adolescents: a systemic review and meta-analysis
Source: BMC Infect Dis. 2019 Aug 30;19:760. doi: 10.1186/s12879-019-4397-1 (PMC6717384; doi:10.1186/s12879-019-4397-1)
Supplement: Supplementary file 3 — Funnel plots for the assessment of publication bias. (DOCX 127 kb) [file 12879_2019_4397_MOESM3_ESM.docx]

| Presepsin | CRP | Procalcitonin |
| --- | --- | --- |
| Egger’s test p-value = 0.0001 | Egger’s test p-value = 0.2812 | Egger’s test p-value = 0.0561 |
| 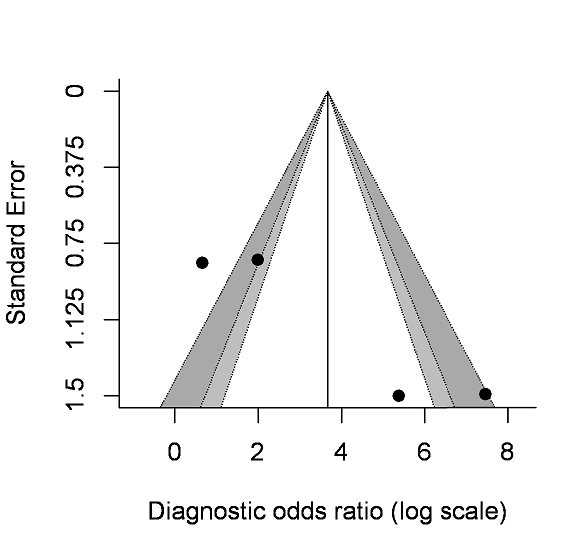 | 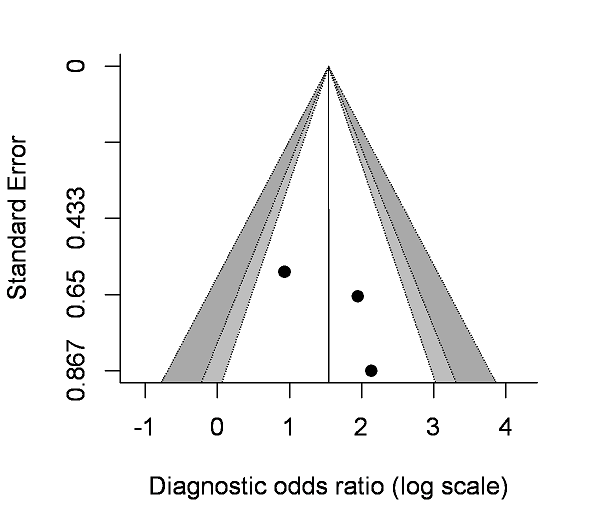 | 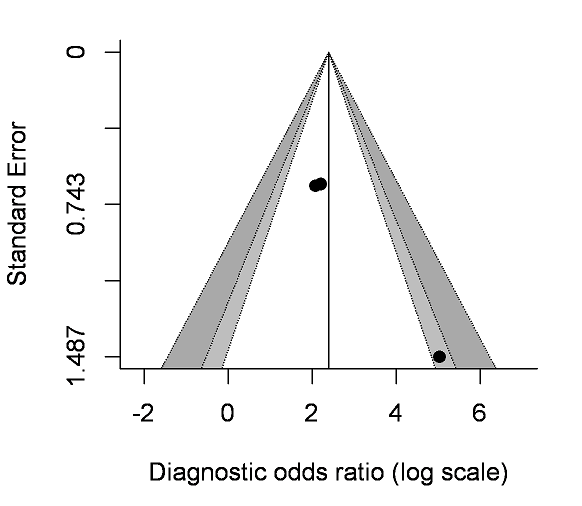 |

Additional File 3. Log (DOR) are 3.55 for presepsin (unadjusted), 1.54 for CRP (C-reactive protein), and 2.29 for PCT (procalcitonin). Egger’s test revealed a publication bias in the studies for presepsin (*P* < 0.05) but not in those for CRP or PCT.
