# Supplementary material for: Presepsin as a diagnostic marker of sepsis in children and adolescents: a systemic review and meta-analysis
Source: BMC Infect Dis. 2019 Aug 30;19:760. doi: 10.1186/s12879-019-4397-1 (PMC6717384; doi:10.1186/s12879-019-4397-1)
Supplement: Supplementary file 4 — Inter-study heterogeneity (subgroup analysis according to the cut-off value). (DOCX 19 kb) [file 12879_2019_4397_MOESM4_ESM.docx]

**Additional File 4. Inter-study heterogeneity.**

|  | Number of studies | Heterogeneity | |
| --- | --- | --- | --- |
|  |  | Sensitivity | Specificity |
| Cutoff ≤ 650 pg/ml | ^2^ | $\chi^{2}=0.00,$p-value > 0.999 | $\chi^{2}=6.29,$p-value = 0.0121 |
| Cutoff > 650 pg/ml | ^2^ | $\chi^{2}=0.00$, p-value > 0.999 | $\chi^{2}=1.38$, p-value = 0.2410 |
| Total | ^4^ | $\chi^{2}=11.17$, p-value = 0.0108 | $\chi^{2}=65.78$, p-value < 0.0001 |

*P* < 0.10 for χ^2^ test indicates substantial heterogeneity.
